# Chitinolytic Bacteria-Assisted Conversion of Squid Pen and Its Effect on Dyes and Pigments Adsorption

**DOI:** 10.3390/md13084576

**Published:** 2015-07-23

**Authors:** Tzu-Wen Liang, Bo-Chang Lo, San-Lang Wang

**Affiliations:** 1 Life Science Development Center, Tamkang University, No. 151, Yingchuan Rd., Tamsui, New Taipei City 25137, Taiwan; E-Mail: ltw27@ms55.hinet.net; 2 Department of Chemistry, Tamkang University, New Taipei City 25137, Taiwan; E-Mail: bunny910817@yahoo.com.tw

**Keywords:** chitosanase, squid pen, *Bacillus cereus*, chitooligomers, biosorbent, adsorption

## Abstract

The aim of this work was to produce chitosanase by fermenting from squid pen, and recover the fermented squid pen for dye removal by adsorption. One chitosanase induced from squid pen powder (SPP)-containing medium by *Bacillus cereus* TKU034 was purified in high purification fold (441) and high yield of activity recovery (51%) by ammonium sulfate precipitation and combined column chromatography. The SDS-PAGE results showed its molecular mass to be around 43 kDa. The TKU034 chitosanase used for the chitooligomers preparation was studied. The enzyme products revealed that the chitosanase could degrade chitosan with various degrees of polymerization, ranging from 3 to 9, as well as the chitosanase in an endolytic manner. Besides, the fermented SPP was recovered and displayed a better adsorption rate (up to 99.5%) for the disperse dyes (red, yellow, blue, and black) than the water-soluble food colorants, Allura Red AC (R40) and Tartrazine (Y4). The adsorbed R40 on the unfermented SPP and the fermented SPP was eluted by distilled water and 1 M NaOH to confirm the dye adsorption mechanism. The fermented SPP had a slightly higher adsorption capacity than the unfermented, and elution of the dye from the fermented SPP was easier than from the unfermented. The main dye adsorption mechanism of fermented SPP was physical adsorption, while the adsorption mechanism of unfermented SPP was chemical adsorption.

## 1. Introduction

Chitosanases are generally endo-splitting enzymes that catalyze the hydrolysis of the β-1,4 glycosidic bonds of chitosan. These enzymes have been found in abundance in a variety of bacteria including *Bacillus* sp. [1,2,3,4], *Serratia* sp. [5], *Janthinobacterium* sp. [6], *Paenibacillus* sp. [7], *Pseudomonas* sp*.* [8], *Acinetobacter* sp. [9] and *Streptomyces* sp. [10]. However, most of the chitosanase-producing bacteria require chitosan as a major carbon/nitrogen source and chitosanase-inducer [1,3,4,6,7,10]. Chitosan has been produced on an industrial scale by the *N*-deacetylation of chitin using sodium hydroxide [11,12]. Among the natural chitinous resources, fishery wastes (shrimp/crab shells and squid pens) have an especially high content of chitin. Therefore, organisms that produce chitosanase with fishery wastes as the sole C/N source can not only solve an environmental problem but can also decrease the production costs of microbial chitosanase. Besides, microorganisms that produce chitosanases without chitosan as an inducer also have technical advantages during fermentation because chitosan has high viscosity and reacts with other compounds in the medium during heat sterilization [13].

Recent researches on chitosanases have received much attention due to their wide range of applications in various fields. One of the most practical and promising applications of chitosanases is the preparation of bioactive chitooligomers (COS). These oligomers have been shown to have potential medical applications, including antitumor, antioxidant [14], and antimicrobial activities [15,16,17,18]. In addition, such functional properties of COS are strongly dependent upon its molecular weight, pentamers and hexamers are particularly active [19]. Chitosanase mediated hydrolysis has advantages over the chemical/physical mediated hydrolytic production of COS, in which chitosanases can catalyze the hydrolysis under mild reaction conditions and do not produce monosaccharides. In addition, it is also the more environmentally friendly process that produces the more defined COS mixtures. Therefore, obtaining an efficient mode for chitosanase production and the conversion of chitosan into bioactive COS would be highly beneficial for producing these oligomeric chitosans efficiently.

For dye removal, most commercial systems currently use activated carbon as sorbent because of its excellent adsorption ability, but it is expensive and difficult to regenerate [20,21]. Recently, researches on the utilization of natural and waste materials for dye removal have also been directed towards alternative adsorbents, namely low-cost adsorbents. Chitin and chitosan have the potential to be inexpensive and effective adsorbents for dye removal [22,23,24]. In recent years, chitin and chitosan have been developed as cheap and effective new alternatives for dye removal. They are also renewable resources and more environmental friendly than commercial materials. In a previous report, *L. paracasei* and *S. marcescens* were used for the production of chitin from crab shell waste by successive fermentation [25]. Consequently, to decrease the cost of chitinous adsorbents for dye removal, the fermented fishery wastes in the culture broth should also be able to be recovered for biological applications in dye removal. This idea interested us, to screen for chitosanolytic enzymes from fishery wastes and reclaim them for dye removal.

In an attempt to screen chitosanolytic enzyme which is capable of converting chitosan into big size-oligomeric chitosan, a new bacterial strain with chitosan degrading activity was sought. A *Bacillus cereus* strain TKU034 that was capable of utilizing SPP to produce chitosanase with high yield was isolated from soil samples. The TKU034 chitosanase was purified, and its biochemical features were also characterized. In addition, the applications of the endo-type TKU034 chitosanase in functional chitooligomer production were also examined. To decrease the cost of adsorbents for dye removal, the fermented SPP was recovered for biological applications in dye removal. The comparisons of the adsorption rates of the fermented SPP and the unfermented SPP for water-soluble food colorants (Allura Red AC, R40; and Tartrazne, Y4) and the disperse dyes (hydrophobic pigments, red, yellow, blue, and black) were also undertaken.

## 2. Results and Discussion

### 2.1. Isolation and Identification of a Chitosanase-Producing Strain

The microorganisms were isolated from soil samples using the procedure described above. A single colony was selected and a bacterial strain tentatively designated as TKU034 producing chitosanase was isolated, and investigated to estimate chitosanase activity on depolymerizing chitosan. The TKU034 strain that showed the highest chitosanase activity among over 200 strains isolated in the laboratory was selected for further study. Strain TKU034 is a Gram-positive and endospore-forming bacillus that contains catalase but not oxidase and is capable of growing in both aerobic and anaerobic environments. It was identified as *Bacillus cereus* species, based on 16S rDNA partial nucleotide sequence (approximately 1.5 kbp) analysis, PCR and homology search for phylogenic tree analysis [26]. According to the API identification, strain TKU034 was the closest to *B. cereus* with a 99.9% similarity. Therefore, the isolate was identified as *B. cereus.*

### 2.2. Preparation and Purification of TKU034 Chitosanase

**Figure 1:**
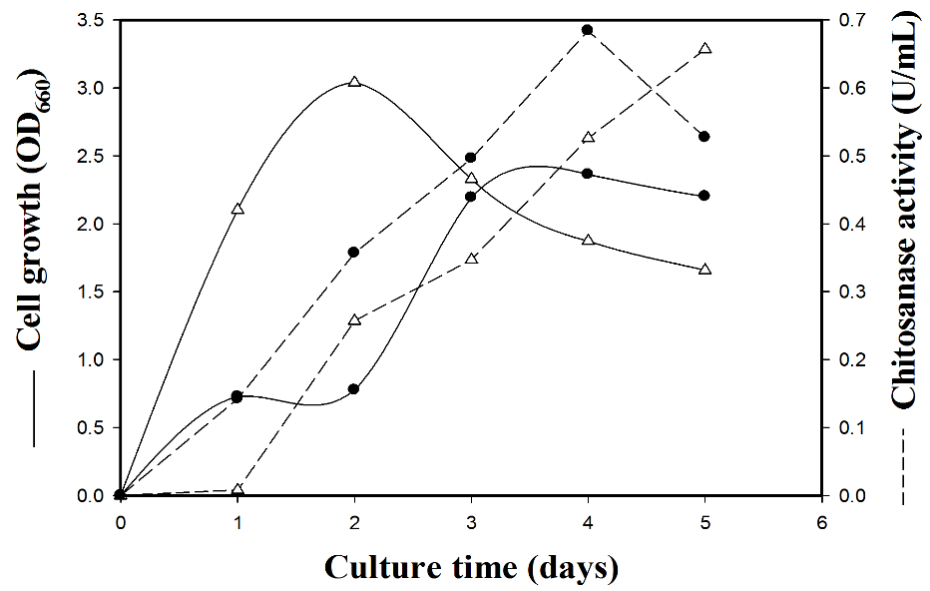
Time courses of chitosanase production from *B.* *cereus* TKU034 in squid pen containing media (●) and nutrient broth (NB) (△).

As shown in Figure 1, the production of chitosanase by strain TKU034 was investigated over five days of cultivation in the production medium. The 100 mL of basal medium (0.1% K_2_HPO_4_ and 0.05% MgSO_4_·7H_2_O, pH 7) containing 1% SPP was the most suitable medium for the production of chitosanase by strain TKU034 at 37 °C. Compared with nutrient broth (NB), the SPP medium increased the enzyme production at the fourth day (Figure 1). The culture supernatant obtained from the bacterial culture in the presence of water-soluble chitosan as substrate displayed gradual chitosan degrading activity that dramatically showed increasing by-products up to four days of cultivation. Exponential growth of *B. cereus* TKU034 was observed for three days, and the stationary phase was reached at the fourth day. The highest chitosanase activity of *B. cereus* TKU034 was detected in the culture on the fourth day of bacterial growth (Figure 1). It was observed that the culture supernatant exerted strong chitosan degrading activities. Conclusively, the results suggested that the chitosanase from *B. cereus* TKU034 may be secreted extracellularly.

**Figure 2:**
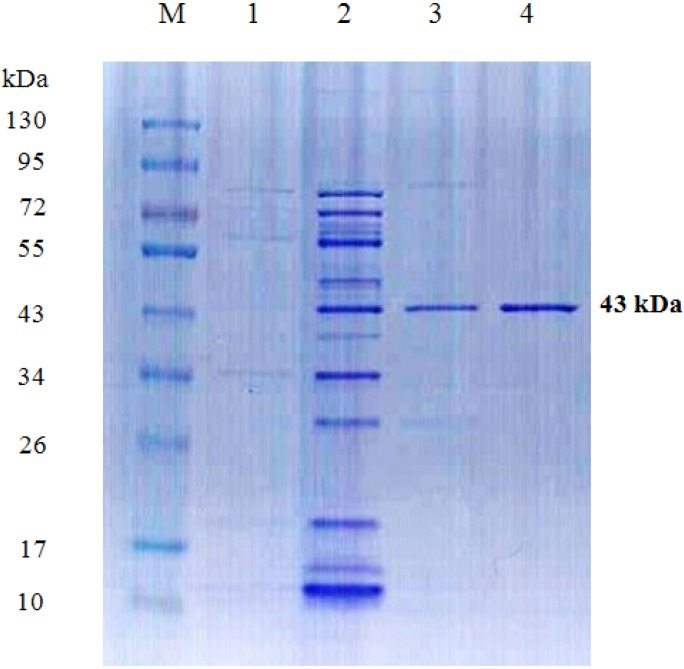
SDS-PAGE analysis of the chitosanase produced by *B.* *cereus* TKU034. Lanes: M, molecular markers (130, 95, 72, 55, 43, 34, 26, 17, and 10 kDa); **1**, culture supernatant; **2**, crude enzyme; **3**, adsorbed chitosanase fractions after DEAE-Sepharose CL-6B chromatography; **4**, adsorbed chitosanase fractions after Macro-prep DEAE chromatography.

An extracellular chitosanase was purified from the cell free culture filtrate of *B. cereus* TKU034 using a series of purification procedures. The TKU034 chitosanase was eluted in the DEAE-Sepharose CL-6B chromatography step with a linear gradient of 0–1 M NaCl in the same buffer. The eluted peak fractions containing the highest chitosanase activities were pooled for further purification. After the Macro-prep DEAE chromatography step, approximately 3.95 mg of TKU034 chitosanase was obtained (Table 1). A summary of the purification process is presented in Table 1. The purification steps were combined to give approximately an overall 441-fold purification of the TKU034 chitosanase. The overall TKU034 chitosanase activity yield was 50.9% with a specific activity of 57.33 U/mg. The molecular mass of the TKU034 chitosanase was approximately 43 kDa as confirmed by the SDS-PAGE (Figure 2), which corresponded to the gel-filtration chromatography. The molecular mass of the TKU034 chitosanase (43 kDa) was similar to most chitosanases, which have a medium apparent molecular mass within the range of 20–75 kDa [13,26], but bacterial ones, especially *Bacillus* species, are usually between 40 and 50 kDa [1,2,26,27,28,29,30]. In our previous study, a microbial chitosanase was reported from *B. cereus* TKU033 using SPP as the sole carbon/nitrogen source, it showed also a molecular weight of around 43 kDa [31]. It is worth comparing the chitosanase activities with the same measure method to provide more valuable aspects on their enzyme activity and purification efficiency. As shown in Table 2, the yield and purification efficiency of TKU034 chitosanase were far higher than those of TKU033 chitosanase. It is expected that TKU034 chitosanase production could be scaled up to meet industrial demand.

**Table 1:** Purification of the chitosanase from *B.* *cereus* TKU034 ^a^.

Step	Total			Specific Activity (U/mg)	Purification (fold)	Recovery (%)
	Volume (mL)	Protein (mg)	Activity (U)			
Culture supernatant	730	3526	445	0.13	1	100
(NH_4_)_2_SO_4_ preciptation	14.5	127.8	440	3.44	26.5	98.9
DEAE-sepharose	38	32.87	411.3	12.51	96.2	92.4
Marcro-Prep DEAE	38	3.95	226.4	57.33	441.0	50.9

^a.^ *B.* *cereus* TKU034 was grown in 100 mL of liquid medium in an Erlenmeyer flask (250 mL) containing 1% SPP, 0.1% K_2_HPO_4_, and 0.05% MgSO_4_·7H_2_O in a shaking incubator for 4 days at 37 °C.

**Table 2:** Comparison of chitosanase activity and purification efficiency between *B.* *cereus* TKU034 and *B.* *cereus* TKU033.

Strains	Culture Supernatant				Final Purification		
	Volume (mL)	Protein (mg)	Activity (U)	Activity (U/mL)	Specific Activity (U/mg)	Purification (fold)	Recovery (%)
TKU034	730	3526	445	0.610	57.33	441	50.9
TKU033	450	4032	19.4	0.043	0.052	10.4	2

### 2.3. Identification of TKU034 Chitosanase by LC-MS/MS Analysis

To identify the protein (TKU034 chitosanase) appearing as prominent 43 kDa band on SDS-PAGE gel, the band was excised and analyzed after tryptic digestion. The band from SDS-PAGE gel was subjected to electrospray tandem mass spectrometry analysis. The fragment spectra were subjected for the NCBI non-redundant protein database search. As shown in Table 3, the spectra of TKU034 chitosanase matched eleven tryptic peptides that were identical to chitosanase from *B. cereus* (GenBank accession number gi33469499, gi52142820, gi446936364, and gi487917180) and *B. thuringiensis* (GenBank accession number gi134143274 and gi446936363) with 33% sequence coverage, and others remaining unmatched. The peptide sequences indicate that the TKU034 chitosanase belongs to the family 8 glycosyl hydrosylase, based on the amino acid sequence similarity of the cited GH-8 enzymes from *B. cereus* and *B. thuringiensis*.

**Table 3:** Identification of the chitosanase from *B.* *cereus* TKU034 by LC-MS/MS.

Peptide Sequence	Identified Protein and Coverage Rate	Accession Number	
^93^NDLSSLPGGYYVK^105^ ^144^IYDGLFK^150^ ^157^SSQNPNLMGWVVADSK^172^ ^173^KAQGHFDSATDGDLDIAYSLLLAHK^197^ ^198^QWGSNGAVNYLK^209^ ^230^LNLGDWDSK^238^ ^239^SSLDTRPSDWMMSHLR^254^ ^255^AFYEFTGDK^263^ ^283^YSPNTGLISDFVVK^296^ ^305^DFLNESEYTNAYYYNASR^322^ ^327^IVMDYAMYGEK^377^	Chitosanase 33%	*Bacillus cereus:* gi|33469499 gi|52142820 gi|446936364 gi|487917180 *Bacillus thuringiensis*: gi|134143274 gi|446936363

### 2.4. Effect of Temperature and pH

**Figure 3:**
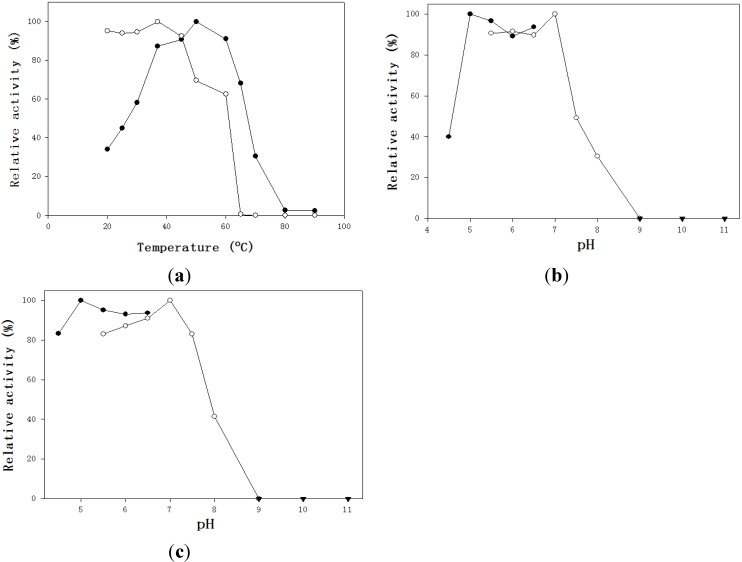
Effects of temperature and pH on the activity and stability of the chitosanase. **(a**) optimum temperature (●) and thermal stability (○); (**b**,**c**) optimum pH (**b**) and pH stability (**c**), (●, acetate buffer; ○, phosphate buffer; ▼, sodium bicarbonate buffer).

The effect of temperature and pH on the chitosanase from *B. cereus* TKU034 was investigated. For the effect of temperature on activity, the purified chitosanase from *B. cereus* TKU034 was active in the range 30–65 °C, being most active at 50 °C. It retained 68% of its highest activity at 65 °C (Figure 3a).

The effect of temperature on stabilty was investigated by measuring residual activity after preincubating the enzymes at different temperatures for 60 min. More than 90% of the initial activities were retained after incubation at 20, 25, 30, 40, and 45 °C (Figure 3a). Less than 10% of the residual activity could be detected after incubation at 65 °C, but it was completely inactivated at 70 °C (Figure 3a).

The TKU034 chitosanase had 90% of its activity at 60 °C. However, when the enzyme was incubated for 60 min at 60 °C and pH 7, in the presence of 0.3% (w/v) water-soluble chitosan, the residual activity of the enzyme was as much as 60% (data not shown). These results indicated that the substrate could prevent thermal inactivation of the chitosanase activity. Enhancement of chitosanase thermostability by the substrate has previously been reported for chitosanase of the GH family 8 from *Bacillus thuringienesis* [3] and the GH family 46 from *Bacillus subtilis* 168 [4].

The effects of pH on enzyme activity and stability were analyzed, and showed in Figure 3b and Figure 3c. The pH activity profiles of the TKU034 chitosanase revealed maximum activity at pH 5 and pH 7 (Figure 3b). The optimum pH (pH 5) for TKU034 chitosanase activity was similar to that of most bacterial chitosanases, which display optimum activities at acidic pH values in a range from 4 to 7 [1,2,10,26,27,28]. The pH stability profiles of the TKU034 chitosanase were determined by measurement of the residual activity at pH 7 after incubation at various pH values at 37 °C for 60 min. The chitosanase was relatively stable at pH 4–7.5 and retained more than 83% of the initial activity in that range (Figure 3c). The TKU034 chitosanase became more sensitive to pH changes above pH 8. The decrease of activity at higher pH ranges may be due to the instability of the protein, rather than an acid-base catalytic mechanism, as shown in previous results [10,32].

### 2.5. Substrate Specificity

For the substrate specificity of the TKU034 chitosanase, chitin and chitosan with DD ranging from 60% to 98% were used as the substrates in 50 mM phosphate buffer, pH 7. The highest activity found was in the presence of water-soluble chitosan (Table 4), although some detectable activity was observed against other substrates. However, these activities are not considered significant compared with that of the chitosanase activity. It could not hydrolyze colloidal chitin, chitosan with DD ranging from 60% to 95% or CMC, but decomposed 98% DD chitosan, chitosan (Mw 140,000–220,000) and chitosan (Mw 110,000–150,000) at 15%, 8%, and 34% of the activity of water-soluble chitosan, respectively. In previous reports, most bacterial chitosanases hydrolyze chitosan efficiently and chitin to a lesser extent [10]. These results indicate that the physical form of the substrate affects the rate of hydrolysis. This is probably due to evolutionary adaption, both to the available natural substrates encountered in their environments and to different key functions within their hosts. However, fungal chitosanases have shown no detectable activity towards chitinous substrates [1,33]. Taken together, these results suggest that the TKU034 chitosanase produced in a specific manner from *B. cereus* TKU034 may be a useful tool for the chitosan biotechnological industry in the production of COS.

**Table 4:** Substrate specificities of the chitosanase from *B. cereus* TKU034.

Substrate	Relative Activity (%) *
Water-soluble chitosan	100
Colloidal chitin from SPP	0
Suspended chitin α	1.2
Suspended chitin β	1.2
Suspended chitin from shrimp shells	0
Colloidal chitin azure	0
Suspended chitosan (98% DD, α-type)	15
Suspended chitosan (98% DD, β-type)	15
Suspended chitosan (95% DD)	0
Suspended chitosan (80% DD)	0
Suspended chitosan (60% DD)	0
Suspended chitosan from shrimp shells	7
Suspended chitosan high purity Mw 60,000–120,000	0
Suspended chitosan high purity Mw 140,000–220,000	8
Suspended chitosan high purity Mw 110,000–150,000	34
Soluble CMC	0

* The relative activity of the chitosanase: 100% = 2.74 U/mL.

### 2.6. Effects of Surfactants and Metal Ions

For the effects of metal ions on enzyme activity, the TKU034 chitosanase activity was measured in response to different metal ions and it was demonstrated that preincubating the enzyme with 5 mM Fe^2+^, Ca^2+^, Cu^2+^, Zn^2+^, or Mn^2+^ resulted in 56%, 74%, 100%, 70%, and 78% inhibition, respectively and EDTA as chelator in the reaction mixture also decreased the enzyme activity (Table 5). However, the TKU034 chitosanase activity was not affected by phenylmethanesulfonyl fluoride (PMSF). Thus, PMSF could be used to control the crude extract serine protease activity and prevent exhaustive protein degradation during downstream processes. Furthermore, the effects of different surfactants (SDS, Tween 20, Tween 40, and Triton X-100) on the stability of the TKU034 chitosanase were also studied. A high stability was observed for the TKU034 chitosanase towards various surfactants (Table 5). Upon incubation with 0.5%–2% of Tween 20, Tween 40, and Triton X-100, the TKU034 chitosanase activity increased slightly with the concentrations of these surfactants. These results may be based on the fact that surface-active reagents might increase the turnover number of chitosanases by increasing the contact frequency between the enzyme active site and the substrate, which is accomplished by lowering the surface tension of the aqueous solution. However, in the presence of 2 mM SDS, the TKU034 chitosanase activity retained 84% of its original activity level. These results suggested that the disulfide bond in the enzyme molecule is associated with its chitosanolytic activity.

**Table 5:** Effects of various chemicals and surfactants on the activities of the chitosanase from *B. cereus* TKU034.

Chemicals	Concentration (mM)	Relative Activity (%)
None	0	100
Mg^2+^	5	91
Fe^2+^	5	44
Ca^2+^	5	26
Cu^2+^	5	0
Ba^2+^	5	93
Zn^2+^	5	30
Mn^2+^	5	22
EDTA	5	59
PMSF	5	111
SDS	0.5/1/2	92/82/84
Tween 20	0.5/1/2 (%)	126/114/112
Tween 40	0.5/1/2 (%)	101/110/109
Triton X-100	0.5/1/2 (%)	115/106/112

Purified enzyme was preincubated with the various reagents at 25 °C for 30 min, and residual chitosanase activity was determined as described in the text. One hundred per cent was assigned to the activity in the absence of reagents. The relative activity of the chitosanase: 100% = 3.30 U/mL.

### 2.7. Enzymatic Digestion of Chitosan by TKU034 Chitosanase

**Figure 4:**
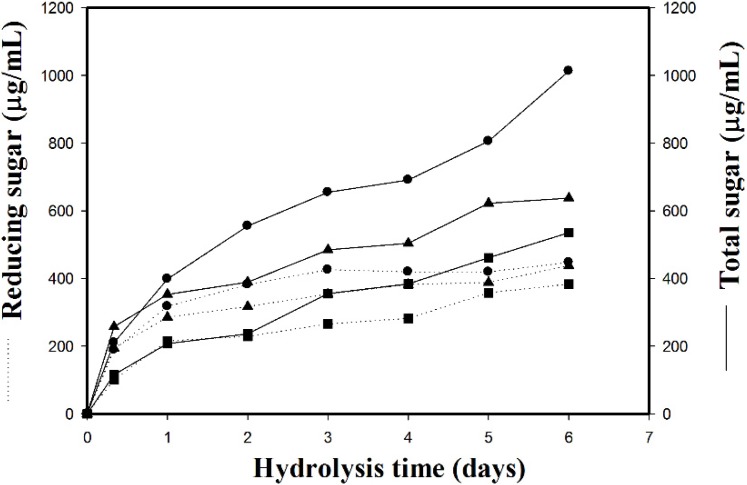
Hydrolysis time course measurement of reducing sugar (···) and total sugar (—) with the chitosanase from *B. cereus* TKU034. Chitosanase activity: ●, 10 U/mL; ▲, 5 U/mL; ■, 1 U/mL.

**Figure 5:**
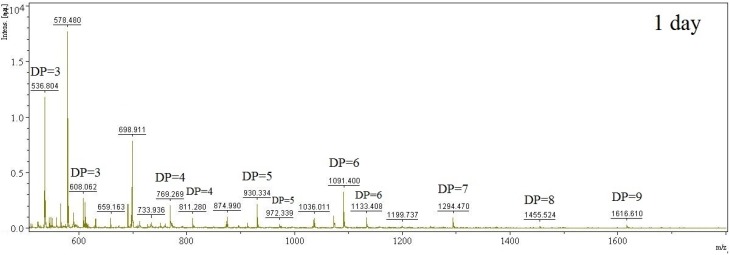
MALDI-TOF-MS of the chitooligomers (COS) mixtures obtained during the chitosan hydrolysis with the TKU034 chitosanase. The proportion of low molecular weight oligomers was reduced by precipitation in the 90% methanol soluble/90% acetone insoluble fraction. The identified peaks are labelled with DP, in which DP indicates the degree of polymerization. The hydrolysis time is labeled in each spectrum.

To evaluate the applicability of the TKU034 chitosanase for the enzymatic digestibility of chitosan into oligosaccharides, the crude enzyme from *B. cereus* TKU034 was used in the experiments. The production system of TKU034 chitosanase was highly efficient and fully available in our laboratory. The chitosan hydrolysis was carried out at 37 °C for six days. The reaction mixture in an Erlenmeyer conical flask (50 mL capacity) contained 0.5% of colloidal chitosan in 20 mL of phosphate buffer (pH 7, 50 mM) and 2.5 mL of concentrated enzyme preparation. Aliquots were withdrawn at different intervals and heated in a boiling water bath for 15 min to terminate the enzyme activity. After removing the undigested material by centrifugation (10,000 rpm, 20 min), the course of chitosan sample degradation was conveniently studied by measurement of the reducing sugars and total sugars [34] in the supernatant. We confirmed that the release of reducing sugars into the supernatant fraction completely ceased after six days of incubation. The amounts of the insoluble materials remaining after the digestion of TKU034 chitosanase were determined and compared with the starting amounts of the chitosan. The amount of insoluble materials remaining was the lowest at the sixth day of digestion with TKU034 chitosanase, indicating that TKU034 chitosanase has 99% digestibility for chitosan (data not shown). As shown in Figure 4, the results revealed the total sugar and the reducing sugar of the sample as a function of reaction time. The total and reducing sugars increased, and the chitosan sample recovery decreased dramatically in the early reaction stage, which can be attributed to an endo-type degradation process. The TKU034 chitosanase was added into the reaction solution after six days and continued hydrolyzing, but it did not improve the increase in total and reducing sugar levels (data not shown). Selective precipitation in 90% methanol and acetone solutions was performed to obtain low DP oligomers, as described earlier [16]. A product analysis of the 90% acetone solution precipitation by MALDI-TOF revealed that various COS had DP values up to 9 (Figure 5). The higher DP chitooligomers were precipitated as a light yellow powder in the methanol solution. The low DP oligomer fraction MALDI-TOF-MS revealed pronounced differences among the crude enzyme-generated chitooligomers, as demonstrated for chitosan depolymerization in Figure 5. The hydrolysate ions present in the mass spectra were identified as sodium adducts [M + Na]^+^. The MALDI-TOF analysis is limited to molecular weights higher than 500 Da because of matrix signal interference; therefore, DP < 2 oligomers could not be determined by this method. After the hydrolysis from the enzymatic reaction over six days, (GlcN)_3_ (*m*/*z* 536), GlcN-(GlcNAc)_2_ (*m*/*z* 608), (GlcN)_2_-(GlcNAc)_2_ (*m*/*z* 769), (GlcN)_3_-(GlcNAc)_2_ (*m*/*z* 930), and (GlcN)_4_-(GlcNAc)_2_ (*m*/*z* 1091) were the major products. In addition, other clear signals (*m*/*z* 685, 727, 811, 972, 1133, 1175, 1294, 1455, and 1616) were also detected (Figure 5). The differences in *m*/*z* values of these signals with those of the corresponding COSs were 42 atomic mass units, which corresponded to the mass weight of acetyl. The hydrolysates contained chitooligomers (GlcN-oligomers) and several partial *N*-acetylated forms. The TKU034 chitosanase reaction product is a mixture of DP 3–9 hetero-chitooligomers. These results indicate that the TKU034 chitosanase might hydrolyze chitosan in an endo-type fashion. From these results, chitosan hydrolysis by the TKU034 chitosanase, combined with a selective methanol precipitation, is a quick and simple method to obtain good chitooligosaccharide yields with DPs up to nine and low molecular weight oligomers.

### 2.8. Dye Removal by Adsorption Processes Using Fermented SPP

**Figure 6:**
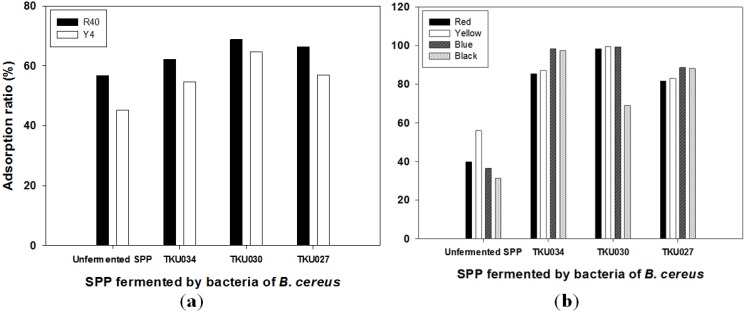
Adsorption effect of squid pen powder (SPP) fermented by bacteria of *B.* *cereus* on food colorants (**a**) and disperse dyes (**b**).

Chitin is known to be an effective adsorbent in the adsorption of dyes [22,23,24]. It was extracted primarily from exoskeleton shellfish by chemical treatments. The costs of chitin-related preparations therefore were far higher than those of their raw materials, marine chitin-containing byproducts. In this study, we chose SPP as the biosorbent for dye removal. For comparison, the 4-day fermented SPPs recovered from the culture broth of chitosanase-producing strains, including *B. cereus* TKU027, *B. cereus* TKU030 and *B. cereus* TKU034 were also analyzed. Appropriate amounts of the reclaimed SPP were added into 2 mL of dye-containing solutions. After shaking at 25 °C for 1 h, the reaction mixtures were centrifuged, and the residual dye in the supernatants was analyzed. The results showed that the color of SPP clearly changed from the colorless original to the color of the dye. The measured adsorption rates for all of the tested dyes also increased with the amounts of the fermented SPP added (data not shown). As shown in Figure 6a and Figure 6b, the adsorption capacities of the fermented SPP on food colorants (Allura Red AC, R40, and Tartrazine, Y4) and disperse dyes (disperse red 60, disperse yellow 54, disperse blue 60 and disperse black 30) were higher than the unfermented SPP. Besides, the fermented SPP exhibited higher adsorption rates (up to 99.5%) on the disperse dyes (hydrophobic pigments) than food colorants (water-soluble pigments). Different results were found in our previous report [24] in which SPP (unfermented) showed a higher adsorption rate for water soluble colorants but a lower adsorption rate for the hydrophobic colorant (prodigiosin) than those of cicada casting and *Lactobacillus* cells. The impact of SPP adsorption for water soluble colorants had been inferred to be related to the protein contained in SPP [24]. However, in this study, we compared the difference of the dye adsorption between the fermented SPP and the unfermented SPP, in addition to the difference of the protein in the two types of SPP; the difference of chitin structure in them should also be considered. Chitin is an amino polymer and has acetamide groups at the C-2 positions. The fermented SPP produced by chitosanase-producing strains, *B. cereus* TKU027, *B. cereus* TKU030, and *B. cereus* TKU034, might result in a number of amino group being accessible on the surface of the fermented SPP. Indeed, the diverse functional groups on chitin and chitosan, such as amino and hydroxyl groups, make it an outstanding dye-binding material. The presence of these groups might be highly advantageous in providing distinctive adsorption functions for the disperse dyes.

### 2.9. Adsorption Mechanism

**Figure 7:**
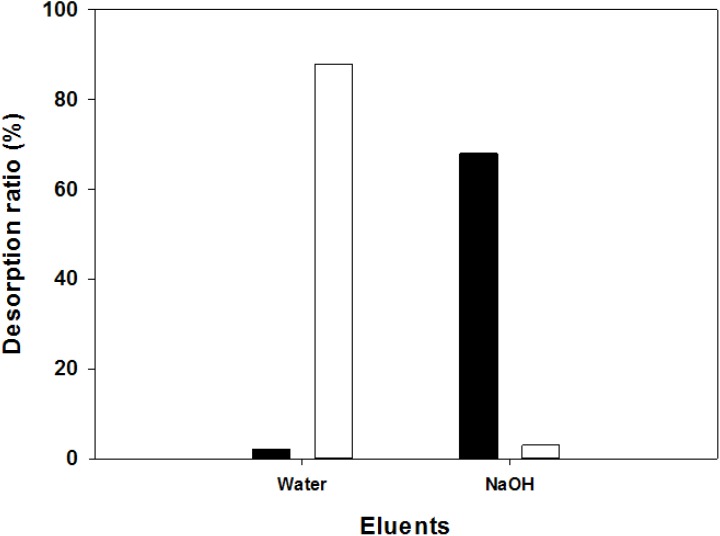
Desorption efficiency of the unfermented SPP (■) and the fermented SPP (□) on Allura Red AC, R40.

After adsorption of Allura Red AC (R40) by unfermented SPP and fermented SPP by *B. cereus* TKU034, the elution test was performed to confirm the dye adsorption mechanism of unfermented SPP and fermented SPP in order to know the nature of the process by which the dye remains adhered to the surface of the adsorbents. Figure 7 indicates that fermented SPP was eluted by distilled water more than unfermented SPP. The result showed that distilled water could elute the dye from unfermented SPP and fermented SPP at 2% and 88%, respectively. This might be due to the modified structure of the fermented SPP. The major parts of SPP are chitin and protein. Chitin is used as an effective adsorbent in the adsorption of dyes. Water can destroy the van der Waals’ force between the azo group (–N=N–), amino group (–NH_2_), and the amide group (–CO–NH–) of chitin. However, there are –SO_3_^−^ groups in the structure of the dye. These groups make the R40 rather acidic. The amino group in the structure of chitin has a great capability to adsorb contaminants due to its Lewis base character. This group is charged positively when chitin is added to aqueous solutions of R40 due to the acidity of the aqueous solution, and thus an electrostatical interaction occurs between this positive charge with the negative charge in the structures of R40. The leaving group (Na) of the dye can undergo nucleophilic displacement by SO_3_^−^ on unfermented SPP, so that the covalent linkage is difficult to destroy with only distilled water. After the elution of water, NaOH is then used as another eluent. This substance may destroy the covalent linkage between the dye and absorbents. The results showed that NaOH could elute the dye out of unfermented SPP and fermented SPP at 68% and 3%, respectively. Therefore, the main dye adsorption mechanism of fermented SPP was physical adsorption, while the adsorption mechanism of unfermented SPP was chemical adsorption. The Fourier transform infrared spectra (FTIR) of the fermented SPP before and after the adsorption of R40 showed no significant change in the adsorption intensity. This also proved that the adsorption process of the fermented SPP was physical adsorption and may not involve a chemical interaction. These results provide a summary of the available information on SPP reclaim and its potential as a low-cost sorbent.

## 3. Materials and Methods

### 3.1. Materials

The squid pens were purchased from Shin-Ma Frozen Food Co. (I-Lan, Taiwan) and used as the sole carbon/nitrogen source for the production of chitosanase by microorganisms. A low molecular weight water-soluble chitosan (5–250 cps nominal viscosity in water at 25 °C, 90% deacetylation degree, DD) from shrimp and crab shell waste was purchased from Charming & Beauty Co. (Taipei, Taiwan). The average particle size was about 106 μm. DEAE-Sepharose CL-6B was purchased from GE healthcare UK Ltd. (Little Chalfont, Buckinghamshire, UK). The weak base anion exchange Macro-prep DEAE was obtained from Bio-Rad (Hercules, CA, USA). Allura Red AC and Tartrazine were obtained from the First Cosmetics Works Company (Taipei, Taiwan). The disperse dyes were obtained from Widetex Biotech Co., Ltd. (Taoyuan, Taiwan). All other reagents used were of the highest grade available.

### 3.2. Screening and Identification of Chitosanase-Producing Strains

The microorganisms were isolated from soil samples collected at different locations in northern Taiwan and screened for chitosan degrading activity after initial cell growth in basal liquid medium (pH 7.2) consisting of 1% SPP, 0.1% K_2_HPO_4_ and 0.05% MgSO_4_·7H_2_O. One gram of soil was ground in a porcelain mortar, 10 mL of sterile distilled water was then added, and the soil suspension was stirred. The soil was eliminated by gentle centrifugation, and the supernatant was centrifuged to harvest the suspended cells. The cell pellet harvested was spread on agar plate containing complete medium to obtain the various heterotrophic bacteria as well as on basal agar medium containing 1% SPP. The organisms obtained from this screening were subcultured in liquid media (containing 1% SPP, 0.1% K_2_HPO_4_, and 0.05% MgSO_4_·7H_2_O) in shaking flasks at 30 °C on a rotary shaker (150 rpm, Yih Der LM-570R). After incubation for 2 days, the culture broth was centrifuged (4 °C at 12,000 g for 20 min, Kubota 5922), and the supernatants were collected for the measurement of chitosanase activity using the procedure described below. The TKU034 strain showed the highest chitosanase activity, and it was isolated, maintained on SPP agar and used throughout the study.

The bacterial strain TKU034 was identified on the basis of morphological, physiological, and biochemical parameters as well as on the basis of a 16S rDNA-based sequence analysis after PCR amplification with primers. The nucleotide bases of the DNA sequence obtained were compiled and compared with sequences in the GenBank databases using the BLAST program. Further identification of the strain TKU034 was performed using the analytical profile index (API).

For API, the strain TKU034 was grown on nutrient agar plates. The bacteria that grew on the surface of the agar plates were suspended by gentle mechanical agitation in 2 mL of sterile distilled water. This bacterial suspension was used to inoculate 50 CHB API strips (ATB system, bioMérieux SA, Marcy-l’Etoile, France) following the manufacturer’s instructions. The strips were incubated at 30 °C and observed after 16, 24, 40, and 48 h and compared to the API identification index and database.

### 3.3. Preparation of Chitosanase

For the production of the chitosanase, *B.* *cereus* TKU034 was grown in 100 mL of liquid medium in an Erlenmeyer flask (250 mL) containing 1% SPP, 0.1% K_2_HPO_4_, and 0.05% MgSO_4_·7H_2_O (pH 7). One mL of the seed culture was transferred into 100 mL of the same medium and grown in an orbital shaking incubator for 4 days at 37 °C and pH 7.2 (the pH after autoclaving was 7.5). After incubation, the culture broth was centrifuged (4 °C at 12,000 g for 20 min), and the supernatant was used for further purification via chromatography.

### 3.4. Chitosanase Activity Assay

Chitosanase activity was assayed using water-soluble chitosan as the substrate directly dissolved in phosphate buffer. The chitosan degrading activity was measured by incubating 0.2 mL of the enzyme solution with 1 mL of 0.3% (w/v) water-soluble chitosan in 50 mM phosphate buffer, pH 7, at 37 °C for 30 min. The reaction was stopped by heating the reaction mixture to 100 °C for 15 min, followed by cooling down the reaction mixture to room temperature over 10 min. The amount of reducing sugar produced was measured with glucosamine as the reference compound [17]; one unit of enzyme activity was defined as the amount of enzyme that produced 1 μmol of reducing sugar per min. The specific activity was expressed as units per mg protein (U/mg protein) of the enzyme extract.

### 3.5. Purification of Chitosanase

The culture supernatants from *B.* *cereus* TKU034 were precipitated and centrifugally collected at 80% saturation of ammonium sulfate precipitation. The precipitates were then dissolved in a small amount of 50 mM sodium phosphate buffer (pH 7) and dialyzed against the same buffer. The resulting dialysate was loaded onto a DEAE-Sepharose CL-6B column (5 cm × 30 cm) that had been equilibrated with 50 mM sodium phosphate buffer (pH 7), washed with the above buffer and eluted with a linear gradient of 0–1 M NaCl-containing buffer. Fractions of 4 mL were collected. The fractions with high chitosanase activities were pooled and concentrated using ammonium sulfate precipitation. The resultant precipitate was collected by centrifugation and dissolved in 5 mL of 50 mM sodium phosphate buffer (pH 7). The concentrates were used for a further chromatography step on a Macro-prep DEAE column (12.6 mm × 40 mm) that had been equilibrated with 50 mM sodium phosphate buffer (pH 7). The chitosanase was eluted using a linear 0–1 M NaCl gradient in the same buffer. Fractions of 1.5 mL were collected. The chitosanase-active fractions were obtained and combined for subsequent characterization.

The protein content was determined using the Bradford method with a Bio-Rad dye reagent concentrate and bovine serum albumin as the standard. After the column chromatography, the protein concentration was estimated by measuring the absorbance at 280 nm [17].

### 3.6. Electrophoresis

Sodium dodecyl sulfate-polyacrylamide gel electrophoresis (SDS-PAGE) was performed according to Laemmli [35] using 12.5% (w/v) polyacrylamide resolving gels and 3.7% (w/v) polyacrylamide stacking gels.

### 3.7. Mass Spectrometry and Protein Identification

The bands of interest were excised from the SDS-PAGE gel and in-gel digested by trypsin. The identity of the enzyme was determined by using liquid chromatography-tandem mass spectrometry (LC-MS/MS) performed by Mission Biotech, Taiwan. The fragment spectra were searched against the NCBI non-redundant protein database. The database searches were performed using the MASCOT search engine.

### 3.8. Effects of Temperature and pH on Enzyme Activity and Stability

The optimum temperature for chitosanase activity was studied by incubating the purified enzyme with substrates at temperatures ranging from 20 °C to 90 °C under standard assay conditions. Thermal stability was determined by pre-incubating the enzyme, without substrate, in sodium phosphate buffer (pH 7) for 60 min at temperatures from 20 °C to 90 °C. The residual activity was measured under standard conditions.

The optimum pH of chitosanase was investigated in the same way as enzyme assay described above, except that reaction buffers had pH values in the range 4.5–11, using 50 mM of each buffer: acetate (pH 4.5–6.5), phosphate (pH 5.5–9) and sodium bicarbonate (pH 9–11). The pH stability of TKU034 chitosanase was determined by measuring the residual activity at pH 7 under standard conditions as described above after the sample had been dialyzed against various pH values in seamless cellulose tubing (Daiichi Sankyo, Tokyo, Japan), without substrates, in the above buffer systems at 4 °C for 30 min. All measurements were repeated at least three times.

### 3.9. Effects of Various Chemicals and Surfactants on Chitosanase Activities

The effects of metal ions (5 mM) were investigated using Mg^2^^+^, Cu^2^^+^, Fe^2+^, Ca^2^^+^, Zn^2^^+^, Mn^2^^+^, and Ba^2+^. The effects of enzyme inhibitors were studied using phenylmethylsulfonyl fluoride (PMSF) and ethylenediaminetetraacetic acid (EDTA). The effects of surfactants were also studied using SDS, Tween 20, Tween 40, and Triton X-100. The enzyme was pre-incubated with various chemicals and surfactants at 4 °C for 30 min, and the residual chitosanase activities were then tested.

### 3.10. Adsorption Experiments of Fermented SPP

The food colorants (R40 and Y4) and disperse dyes (red, yellow, blue, and black) (0.05%, w/v) were added to different concentrations of the fermented and unfermented SPP, respectively, and incubated at 25 °C on a rotary shaker (150 rpm) for one hour. After centrifugation, the residual pigments in the supernatant were then analyzed with a spectrophotometer (the absorption wave lengths of R40, Y4, disperse dyes red, yellow, blue, and black were 504, 425, 530, 415, 555, and 580 nm, respectively). The adsorption ratios were calculated with the formula

Adsorption ratio (%)=(C − E)/C × 100

where C is the absorbance of the control group and E is the absorbance of the experimental group.
